# Supplementary material for: Improved Sugarcane-Based Fermentation Processes by an Industrial Fuel-Ethanol Yeast Strain
Source: J Fungi (Basel). 2023 Jul 29;9(8):803. doi: 10.3390/jof9080803 (PMC10456111; doi:10.3390/jof9080803)
Supplement: Supplementary file 1 [file jof-09-00803-s001.zip › Suplementary Figure S1 revised.pdf]

Improved Sugarcane-based Fermentation Processes by an Industrial Fuel-ethanol Yeast Strain

Gabriela Muller [1], Victor R. de Godoy [1], Marcelo G. Dário [1], Eduarda H. Duval [1], Sergio L. Alves-Jr [1], Augusto Bücker [1], Carlos A. Rosa [2], Barbara Dunn [3], Gavin Sherlock[3] and Boris U. Stambuk [1,*]

[1] Department of Biochemistry, Federal University of Santa Catarina, Florianópolis 88040-900, SC, Brazil;
[2] Departamento de Microbiologia, Universidade Federal de Minas Gerais, Belo Horizonte, Minas Gerais 31270-901, Brazil;
[3] Department of Genetics, Stanford University, Stanford, California 94305, United States of America;
* Correspondence: boris.stambuk@ufsc.br; Tel.: +55 48 99615-9566

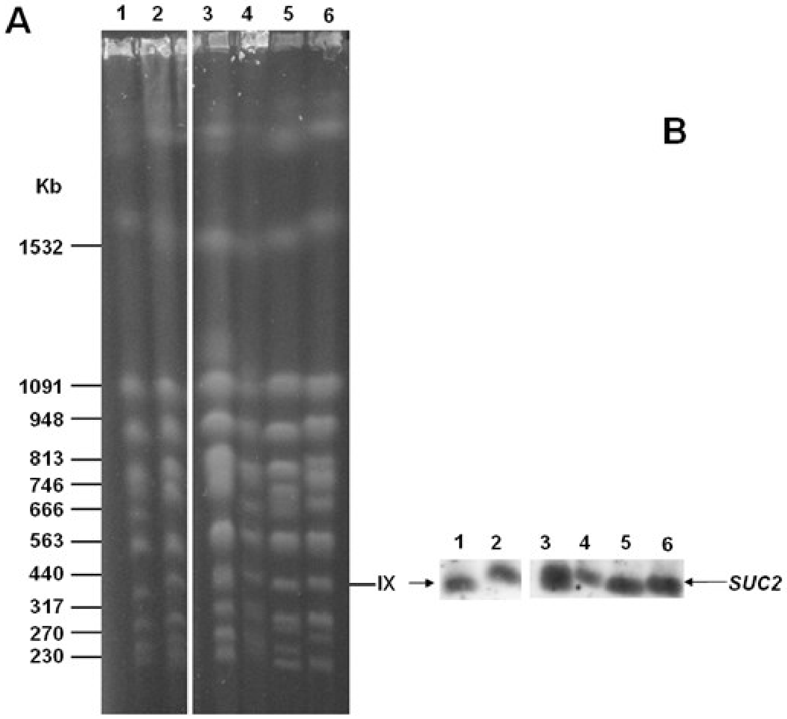

Supplementary Figure S1. Detection of *SUC* genes in additional industrial sugarcane yeasts. (**A**) PFGE separation of yeast chromosomes (ethidium-bromide stained). (**B**) Southern blot of gels shown in panel (**A**), hybridized with a probe for *SUC2* to detect which chromosomes carry *SUC* genes. (Lane 1) Reference laboratory strain S288C, which contains *SUC2* on chromosome IX (indicated to the right of panel **A**); (lanes 2–6) strains CAT-1, UFMG-1007, UFPE-179, PE-2, and VR-1, respectively. The values on the left in panel **A** are the sizes (in kilobase pairs) of selected S288C chromosomes. Besides those represented by the bands shown in panel **B**, no other chromosome hybridized with the *SUC2* probe.
